# A case report and a literature review of double mammary pseudoangiomatous stromal hyperplasia associated with galactoma during pregnancy

**DOI:** 10.3389/fonc.2024.1359886

**Published:** 2024-03-27

**Authors:** Minmin Yu, Guangxi Shi, Zong Gao, Kai Wu, Cuilei Wei, Xiaohong Li, Xiuming Miao

**Affiliations:** ^1^ Pathology Department, Affiliated Hospital of Shandong University of Traditional Chinese Medicine, Jinan, China; ^2^ Breast and Thyroid Surgery Department, Affiliated Hospital of Shandong University of Traditional Chinese Medicine, Jinan, China; ^3^ Neurosurgery Department, Affiliated Hospital of Shandong University of Traditional Chinese Medicine, Jinan, China; ^4^ First Clinical School of Medicine, Shandong University of Traditional Chinese Medicine, Jinan, China

**Keywords:** mammary gland, pseudoangiomatous stromal hyperplasia, pregnancy period, tumor, breast

## Abstract

Pseudoangiomatous stromal hyperplasia (PASH) is a benign interstitial hyperplasia of the breast that usually occurs in premenopausal or perimenopausal women. It is usually characterized by localized lesions or clear boundary masses, and diffuse double breast enlargement is rare. PASH is considered a hormone-dependent disease that is commonly progesterone related. There are no imaging characteristics, and both benign and suspicious malignant signs can be seen. The definitive diagnosis of PASH depends on a pathological diagnosis, and it is necessary to be vigilant in distinguishing between benign and malignant tumors with similar breast histopathology. Here, we report the case of a 23-year-old multipara patient with bilateral diffuse pseudoangiomatous stromal hyperplasia of the breast during pregnancy who presented with macromastia and reviewed the literature to further understand the clinical features, pathological diagnosis, differential diagnosis, treatment and prognosis of pseudoangiomatous stromal hyperplasia of the breast.

## Background

Pseudoangiomatous stromal hyperplasia (PASH) can exist independently and is mainly or entirely composed of stromal cells (1). It is a rare benign lesion of the breast that was first described by Vuitch et al. (1986). The age of onset ranges from 12 to 86 years (2), and it is most common in premenopausal women and less common in perimenopausal women and men. It has also been reported in preadolescent women, patients with immune deficiency and patients taking immunosuppressants (3, 4). The etiology and pathogenesis of PASH are still unclear, and most scholars believe that its pathogenesis is related to hormone dependence. Most clinical cases involve palpable masses, very few of which can grow diffusely to form giant mammary disease. The clinical manifestations and imaging features are nonspecific (4, 5) and are often ignored or missed. A definitive diagnosis of PASH depends on a pathological diagnosis, and clinicians should be vigilant in distinguishing between benign and malignant tumors with histopathology similar to that of other breast tumors. We report a 23-year-old patient with bilateral diffuse pseudoangiomatous stromal hyperplasia of the breast during pregnancy who presented with galactoma. We also reviewed the literature to further understand the clinical features, pathological diagnosis, differential diagnosis, treatment and prognosis of patients with pseudoangiomatous stromal hyperplasia of the breast.

## Clinical data

A 23-year-old female patient who conceived normally for the first time in January 2018 underwent a cesarean section in November and gave birth to a healthy baby girl. During pregnancy, bilateral mammary glands slightly increased in size symmetrically, and there was normal postpartum lactation. Multiple forms of galactostasis occurred during lactation, and after self-mammary physical therapy, lactation resumed, continuing for half a year. The patient became pregnant again in April 2019. At 12 weeks of pregnancy, asymmetry of the bilateral mammary glands increased, bumps were palpated, and occasional pain was felt. No treatment was administered, and the fetus was found to be normal during pregnancy. In February 2020, a healthy baby girl was delivered by cesarean section again. One week after delivery, bilateral breast gland enlargement was obvious, with a small amount of milk secretion, which continued to increase within three months, accompanied by back pain, local skin thickening and pruritus, and no milk secretion. She took the traditional Chinese medicines Rupixiao and Dianshi pills and then remained in stable condition. She came to our hospital on July 21, 2022, and a color ultrasound examination revealed changes in diffuse edema in both breasts (BI-RADS category 3). Breast MRI revealed multiple nodules and masses in both breasts, which were considered inflammatory granulomas. Pathologic results of tumor resection of the breast mass under local anesthesia revealed pseudohemangiomatoid stromal hyperplasia. One month after the operation, both breasts enlarged rapidly and affected the patient’s life. On August 29 of the same year, double breast mass resection under general anesthesia plus bilateral breast reduction and lift was performed at another hospital, and the pathological results were the same as before. Six months after the operation, the patient developed a new breast mass, which grew rapidly, and the breast volume of the patient returned to its initial size, accompanied by thickening, hardening, and redness of the skin under the areola. This preoperative serological examination of female hormones revealed an increase in estradiol of 3834.49 pmol/ml, and the estradiol level reached a normal value after the operation. After full communication between the surgeon and the patient, bilateral subcutaneous gland resection, inverted mastectomy and skin biopsy were performed on July 4, 2023. Postoperative pathology revealed breast pseudohemangiomatoid stromal hyperplasia, epidermal hyperplasia with hyperkeratosis, granulosa thickening and chronic inflammatory cell infiltration in the dermis. No recurrence was found at the 5-month follow-up after the operation.

## Imaging examination

**Figure 1:**
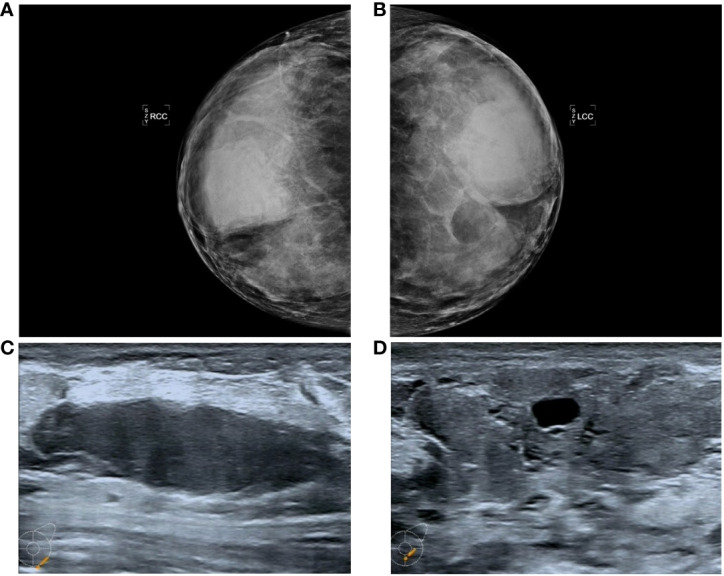
Manifestations **(A, B)** in the PASH image showed changes in the double milk molybdenum target, which showed an oval isondense mass. **(C, D)** show ultrasound changes in both breasts, manifested as hypoechoic nodules with clear boundaries and cystic changes.

Mammography revealed that both mammary glands appeared as lumps of varying size that were elliptic, with borders or partial borders, calcification, or structural distortion (**Figures 1A, B**).

Color ultrasound revealed that the double mammary gland was not clear, the structure was more disordered, the internal echo was not uniform, there were thick spots and light spots, and a patchy irregular sparse area could be seen. CDFI: No important abnormal blood flow in either mammary gland. There were many low-echo and no-echo zones in both breasts. The largest diameter of the no-echo zone was approximately 15.9 mm, and the diameter of the low-echo zone was approximately 19.6-53.9 mm. The shape was regular, the boundary was clear, and the echo was uniform (**Figures 1C, D**).

## Routine pathological examination

### General manifestations

**Figure 2:**
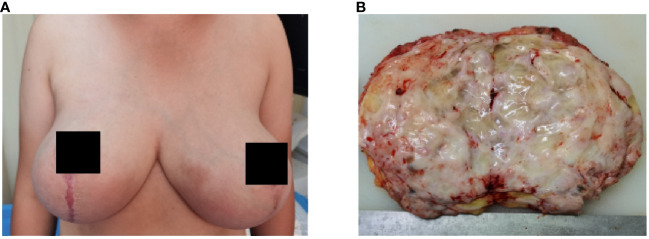
Bilateral mammary gland enlargement in patient **(A)** before surgery; **(B)** gross manifestations of tumor resection.

The left and right mastectomy specimens weighed 5 kg and 5.5 kg, respectively. The sections were multinodular, 0.5-2.5 cm in diameter. The sections were gray and grayish brown, the internodules showed edema or mucous changes, and multiple sac-like structures of different sizes could be seen without bleeding or necrosis (**Figure 2**). The skin tissue of the breast was rough, thickened and pigmented.

### Pathologic features

**Figure 3:**
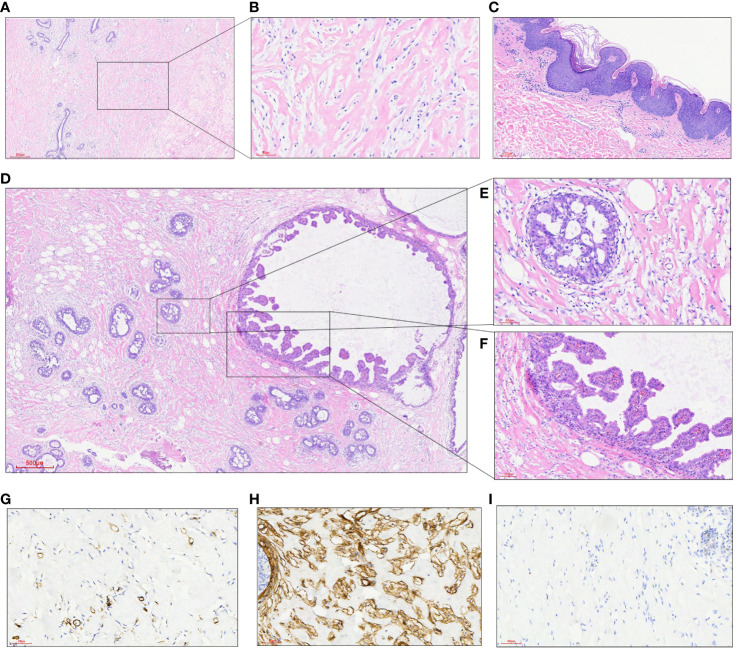
**(A)** At low power, a dense collagen matrix around and within the lobules was observed, with slit-like spaces (HE, 40X); **(B)** there were many fissure-like spaces in the dense collagenous breast interstitium, in which benign spindle cells were arranged without cytological atypia (HE, 200X); **(C)** epidermal hyperplasia of breast skin with hyperkeratosis, granulosa thickening and acanthosis edema; **(D)** focal points showed multiple intraductal common type (UDH) and papillary hyperplasia areas (HE, 40X); **(E)** a local enlarged UDH of the breast (HE, 200X); **(F)** ductal epithelial papillary hyperplasia of the breast (HE, 200X); **(G)** fusiform cells between fissures did not express the vasogenic marker CD31 (200X); **(H)** the expression of CD34 in fusiform cells between fissures was diffusively strong (200X); **(I)** PR was not expressed in fusiform cells of the interface (200X).

The histopathological features under the double mastoscope were similar, as shown by typical PASH images; PASH was diffusely distributed in the interlobular and intralobular tissues of the breast, similar to fibrous scar tissue; irregular fissured spaces of varying sizes were visible; the spaces were lined with mild fusiform cells; and no red blood cells were found in the lacunae. At the same time, there was common ductal epithelial hyperplasia, apocrine metaplasia, columnar cell hyperplasia and cystic changes, and flocculent secretions in the cystic cavity. Luteal phase changes were observed in normal breast lobular units, including lobular-specific interstitial edema, myoepithelial cell vacuolation, and high columnar ductal epithelium with apical plasma secretion. Epidermal hyperplasia of breast skin with hyperkeratosis, granulosa thickening with acanthosis, superficial dermis and perivascular lymphocyte infiltration were observed (**Figures 3A–F**). Immunohistochemical results showed that the fusiform cells of the lacunar lining had positive expression of CD34+, SMA+, CD99+ and BCL-2+, while the vascular and lymphatic endothelial cells labeled with CD31 and D2-40 were negative and Ki-67 was <1%. The duct epithelium had positive expression of ER and PR, and the ER showed a heterogeneous expression pattern with varying intensity. The expression of PR was greater than that of ER, and most of the cells were strongly positive. The fusiform cells of the lacunar lining were negative (**Figures 3G–I**).

## Discussion

PASH is a benign mesenchymal disease of the breast. It is most common in premenopausal or perimenopausal women, and the histopathological features are excessive proliferation of breast interstitial myofibroblasts and the production of rich collagen, which is localized, nodular or diffuse. The clinical manifestations of PASH are localized lesions, which are usually found by chance along with benign and malignant breast diseases. A study by Ibrahim et al. (6) showed that PASH could be found by chance in up to 23% of continuous breast specimens. In addition, 25% of male breast development cases have been reported (7, 8). In contrast, nodular and diffuse PASH, as independent lesions, are rare in clinical practice and typically present as a unilateral, clearly defined mass similar to breast fibroadenoma or lobe tumor, which is more common on one side without nipple or skin changes. A very small number of patients showed diffuse and rapidly enlarging masses on both mammary glands, and only two cases of PASH-related macrosomia in both breasts during pregnancy, accompanied by redness, swelling and orange peel changes, were reported (9, 10). Two previously reported cases of bilateral diffuse mammary pseudoangiomatous stromal hyperplasia during pregnancy presented with macromastia (**Table 1**). The first patient was a 33-year-old dichorionic diamniotic twin at 14 weeks of gestation who presented bilateral breast enlargement in early gestation, accompanied by back pain, limited movement, respiratory disorders and skin damage. The presence of PASH was confirmed by histopathology. Bilateral skin-sparing mastectomy was performed at 16 weeks gestation, and immediate reconstruction was performed. The fetuses were normal throughout the pregnancy, and healthy twins were delivered by cesarean section at 38 weeks gestation. Another 43-year-old primiparous woman presented at week 22 3/7 with bilateral breast enlargement, accompanied by back pain, limited mobility, respiratory disturbance, and skin damage. The patient had a history of a right breast mass on PASH 4 years prior. The patient had attempted to conceive with clomiphene and intrauterine insemination for 3 months and subsequently exhibited significant growth of the right mammary gland, which was pathologically confirmed as PASH. Bilateral mastectomy was performed at 6.5 months of gestation, and the patient delivered naturally to term with a healthy fetus and no other complications. In our case, a 23-year-old female presented with asymmetric enlargement of the bilateral mammary glands at the third month of gestation, with bumps and occasional pain. After delivery, bilateral mammary glands continued to substantially increase in size, without milk secretion, accompanied by local skin thickening and pruritus, and inflammatory granuloma was considered at first diagnosis. Pathology of the tissue from resection under local anesthesia revealed pseudohemangiomatoid stromal hyperplasia of the breast; this development recurred twice within 12 months after local resection, and the patient finally underwent total glandular resection of both breasts. No recurrence was observed in five months of follow-up.

**Table 1:** Overview of the two cases of bilateral breast PASH during pregnancy reported in the literature and this case.

Case	Age	Childbearing		Medical history	Course	Disease time	Clinical feature	Skin changes	Histopathology	Time and method of operation	Follow-up	References
1	33 yr	primigravida		bronchial asthma	initial	at 14 weeks of dichorionic diamniotic twin pregnancy presented	bilateral breast; breast tenderness, back pain with movement limitation and respiratory impairment.	multiple skin ulcerations	typical PASH	bilateral skin-sparing mastectomy at 16 weeks of the pregnancy	no recurrence; healthy twins were delivered by cesarean section at 38 weeks of gestation	(9)
2	43 yr	primigravida		tamoxifen for 3 month; infertility; *in vitro* fertilization (IVF)	recurrence	at 22 3/7 weeks of gestation	bilateral breast; edema throughout her bilateral breasts, bra strap grooving and tenderness with palpation	darkening, moderate erythema and edema	typical PASH	bilateral mastectomy at second trimester of gestation	no recurrence; delivering at term via spontaneous vaginal delivery	(10)
3	23 yr	multipara		multiple galactostasis during first lactation	recurrence	at 12 weeks of gestation	bilateral breast gland enlargement;	thickening and pruritus	typical PASH	bilateral subcutaneous gland resection, inverted mastectomy on July 4, 2023	no recurrence	this case

PASH imaging often shows no specific changes, and both benign and suspicious malignant signs can be observed. It has been reported that nearly 53% of patients with PASH syndrome show abnormalities on screening mammograms (7), and the most common manifestation of PASH syndrome on X-rays is a lump of varying size, usually round or oval, with a border on the edge or a partial border (11). The most common feature of PASH in breast B-ultrasonography is a well-defined low-echo or equal-echo oval mass enhanced by transmission, while the presence of fibrocystic changes can lead to a heterogeneous appearance, generally without calcification (12). Mammography examination mostly revealed round or oval isodense masses with clear boundaries. Polger et al. (13) performed a mammography examination of 7 PASH patients and found isodense masses with a diameter of 1.1-11 cm and no calcification. Breast magnetic resonance imaging (MRI) shows mass or nonmass enhancement and benign dynamic changes (8, 12). In this case, the imaging examination also revealed many low-echo and no echo areas, clear boundaries and uniform echoes.

The histopathological characteristics of PASH can be divided into classic and fascicular types according to the microscopic manifestations. Fissure-like communication branches can be observed in typical PASH, and the inner wall of the fracture is composed of proliferated spindle cells without obvious nuclear atypia or nuclear division. There were no red blood cells in the fissure, and the interwoven collagen fibers exhibited hyaline degeneration. The lesions usually surround the breast lobules, widen the space between the lobules, or extend into the lobules but do not destroy the normal structure of the lobules (2, 14). PASH may be mistaken for low-grade angiosarcoma, which has a true vascular space, on pathological examination (12, 15). Fascicular PASH has more abundant cells, and the fusiform cells in the lesion are arranged in bundles and lack a fissure structure, which is often observed in conjunction with myofibroblastoma (12, 15). In our case, the areas of interlobular and netted interlobular mesenchyme in both breasts were replaced by typical PASH, the lobular units were enlarged or irregular, but no lobular structure was destroyed, and the ductal epithelium was accompanied by exuberant hyperplasia. Common hyperplasia, papillary hyperplasia, columnar cell transformation, cystic dilatation, and flocculent protein secretion can be observed in the cystic dilatation gland lumen, as can nontypical hyperplasia and carcinoma *in situ*. Relatively normal uninvolved lobular units showed luteal phase changes, lobular-specific interstitial edema, myoepithelial cell vacuolation, high columnar ductal epithelium with apical plasma secretion, and no lactation.

The etiology and pathogenesis of PASH remain unclear. Some researchers have combined the histopathological characteristics of PASH and the clinical characteristics of recurrent, multiple and accompanied neoplastic breast lesions, and most scholars believe that its pathogenesis may involve excessive and abnormal responses of breast myofibroblasts to progesterone stimulation (2, 14, 16). Studies have shown that progesterone receptors are highly expressed in PASH stromal cells but not in normal breast stromal cells. Since progesterone is produced by the metabolism of cytochrome P450, drugs metabolized by cytochrome P450, such as clonazepam, sodium valproate and risperidone, increase the level of progesterone and thus stimulate the growth of PASH (16, 17). In this case, the patient presented with galactoma during pregnancy and experienced two recurrences. Histopathology revealed that diffuse PASH in both breasts presented changes in the extent of galactoma changes without structural damage to lobular units, accompanied by vigorous breast ductal epithelium; relatively normal lobular units presented luteal phase changes, lobular specialized interstitial edema, and myoepithelial vacuolation; and the ductal epithelium presented a highly columnar shape with apical plasma secretion. However, no lactation was observed, so the patient could not breastfeed normally after delivery. Combined with the pathogenesis and histopathological findings of the patients, we agree with most scholars that the occurrence of breast PASH is closely related to the increase in progesterone. The occurrence of PASH during pregnancy is due to progesterone secreted by the placenta, which stimulates hyperplasia of mammary stromal myofibroblasts. However, the hypothesis that PASH is associated with elevated hormone levels is controversial. A study by Erin Bowman et al. conducted a retrospective data analysis of 24 patients with PASH and revealed that 95% of the samples were positive for ER or PR, which supported its developmental hormonal basis (12). Interestingly, both the ER and PR were not expressed in the spindle cells of the interstitium in this patient, while both were expressed in the ductal epithelium, with the PR being considerably more highly expressed than the ER. In addition, patients have transient prolactin increases during onset, and whether there is a correlation between them needs to be further confirmed. The pathogenesis of this disease remains to be studied.

Breast PASH needs to be differentiated from low-grade hemangiosarcoma, phyllodes tumor, metaplastic carcinoma with spindle cell differentiation, and hamartoma. Primary hemangiosarcoma of the breast is relatively rare. The tumor is invasive and often has unclear boundaries with surrounding tissues. The histological manifestations are anastomotic branched blood vessels lined with atypical endothelial cells, which express factor VIII, CD31, CD34 and other markers (18). Phyllodes tumors of the breast are biphasic tumors with hyperplasia of the stroma and epithelium of the breast. Hyperplasia of the stroma stimulates the disordered growth of glands, resulting in irregular branching of ducts and distorted lobules and disordered distribution of stroma and glands, forming a blade-like structure, which can be focally combined with PASH. The foliar structure is lined with the ductal epithelium of the breast. The duodenal epithelium expresses glandular epithelial markers (19). Metaplastic carcinoma accompanied by spindle cell differentiation refers to infiltrating adenocarcinoma rich in spindle cells. Spindle cells are heteromorphic and can be nests, sheets or braided, and the cells exhibit certain heteromorphism accompanied by varying amounts of nuclear division; interstitial cells may exhibit varying degrees of collagenization and express the epithelial-labeled spindle cells AE1/AE3, CK7, CK8/18, etc. Breast hamartomas contain varying amounts of fat, fibrous tissue and smooth muscle tissue, and disorganized ducts and lobules can also be observed, possibly accompanied by PASH. The clinical manifestations and imaging findings of PASH in pregnancy overlap with those of granulomatous lobular mastitis (GLM) in the mass stage, which is easily misdiagnosed as GLM and needs to be confirmed by pathological examination.

There are currently no standards for the treatment of PASH. Different treatment options are often selected according to different clinical manifestations. For local lesions, clinicians often choose observation, follow-up, and regular monitoring. Among the principles for the management of benign breast diseases announced at the 19th Annual Meeting of the American College of Breast Surgeons in 2018, the first is that the breast PASH region of asymptomatic patients is removed by irregular surgery (20). Those with nodular lesions > 2 cm need complete resection, and for those with small volumes, follow-up is recommended (12, 21, 22). When nodular PASH presents as macromastia, which affects its appearance, or when the tumor increases rapidly in a short period and it is difficult to determine whether it is benign or malignant, surgical resection is often the first choice of treatment. Ryu et al. (23) reported that patients with galactoma could be treated with breast reduction surgery, and total resection was generally not considered. The patient only underwent local resection of the breast mass at the first visit, and the patient relapsed twice within 11 months after local resection; finally, she underwent total glandular resection of both breasts. The patient recovered well after surgery, and no recurrence was observed at the 4-month follow-up. The recurrence rate of PASH after resection can reach 28.5%, and follow-up is still needed after resection (24). Regarding the timing of surgery for PASH during pregnancy, Jennifer Wang et al. suggested that the best time for surgical intervention is in the early second trimester, after fetal organogenesis has been completed, when the uterine floor is located below the sacral point. It is likely that pregnant women who undergo mastectomy due to PASH-induced mastectomy in the second trimester will recover quickly from an obstetrical point of view, and the risk to the fetus is low. We believe that surgical total mastectomy to prevent the recurrence of PASH-related giant mastectomy is a better treatment. Several researchers have reported a possible role for antihormone therapy in the treatment of breast PASH, but there are insufficient data to demonstrate the effectiveness of such medical management (10, 25, 26).

The prognosis of PASH is good, and no cases of Pash-related death have been reported (3, 16, 26). Drinka et al. (27) confirmed that PASH can accompany invasive breast cancer, but there is no evidence that PASH can promote the occurrence or progression of breast cancer. Degnim (28) reviewed the biopsies of 9065 women who had benign mastectomy biopsies between 1967 and 1991 and estimated the relative risk of subsequent breast cancer with PASH using the standardized incidence ratio (SIR), finding that the presence of PASH did not imply an increased risk of subsequent breast cancer compared with the general population (27).

## Data availability statement

The original contributions presented in the study are included in the article/supplementary material. Further inquiries can be directed to the corresponding author.

## Ethics statement

Written informed consent was obtained from the individual(s) for the publication of any potentially identifiable images or data included in this article.

## Author contributions

MY: Writing – original draft, Writing – review & editing. GS: Writing – original draft, Writing – review & editing. ZG: Writing – original draft, Writing – review & editing. KW: Writing – original draft, Writing – review & editing. CW: Writing – original draft, Writing – review & editing. XL: Writing – original draft, Writing – review & editing. XM: Writing – original draft, Writing – review & editing.
